# Lectures on the Diagnosis and Treatment of Diseases of the Lungs

**Published:** 1841-04-24

**Authors:** W. W. Gerhard


MEDICAL EXAMINER.

DEVOTED TO MEDICINE, SURGERY, AND THE COLLATERAL SCIENCES.

No. 17.] PHILADELPHIA, SATURDAY, APRIL 24, 1841. [Vol. IV.

### LECTURES ON THE DIAGNOSIS AND TREATMENT OF DISEASES OF THE LUNGS.

BY W. W. GERHARD, M. D.

### LECTURE XIII.—(*Continued.*)

#### PHTHISIS—TREATMENT.

The treatment specially intended to prevent the growth of tubercles being very limited in its action, we are obliged to resort to collateral measures, which act rather upon the intercurrent diseases which favour the development of tubercle, than upon this product itself. These intercurrent diseases are for the most part of an inflammatory character, and, as already mentioned, precede or accompany the tuberculous deposit. Those which precede it do not require any special treatment; but we must watch their termination, for the greatest danger is to be feared just at their close. Hence, in long-protracted inflammations of the larynx, trachea, or bronchial tubes, and in severe or repeated attacks of pleurisy, the treatment should be continued until the disease entirely disappears, and the restoration of the patient to his former health is complete.

The intercurrent inflammations of the chest are more difficult to manage, because the tuberculous matter is actually developed, and the directly antiphlogistic treatment must be less continued. Of these inflammations the most common is that of the bronchial or tracheal mucous membranes; most of the tickling and cough depends upon this, and a large portion of the sputa comes from the same source; hence, the patient is solicitous to quiet the irritation, and the physician is constantly tempted to resort to opiates and other palliatives. It is often necessary to give opiates,—that is, if the patient is unable to sleep, the cough must be allayed; and if the tickling and irritation be incessant during the day, it is necessary to quiet them. Still opiates are essentially disadvantageous, and in most cases they should only be resorted to if other means fail, and then in small doses, so as to preserve the stomach in as healthy a condition as possible. Hyosciamus is preferable with many patients to opium; that is, if it tranquillizes the cough—for it will not do so always; a mixture may be made of an ounce of the syrup of Tolu, the same quantity of the syrup of senega, and six grains of hyosciamus, with four ounces of gum arabic mucilage. Half an ounce of wine of ipecacuanha may be advantageously substituted for the syrup of senega in many cases, especially if there be much fever. This quantity may be taken in the course of three days, a dessert spoonful at a time. In this dose it is well to watch the effects of the hyosciamus, for at the end of one or two weeks it will occasionally produce some symptoms of narcotism. The opiates most used are the salts of morphia and the elixir of paregoric; on the whole, the former are preferable, but the quantity should not exceed a quarter or a third of a grain in the twenty-four hours: with many patients it is best to give a small quantity of morphia at night, without any other substance, but in most instances the cough is soothed by a combination of a mucilaginous vehicle and of an expectorant. As expectorants, we may use the syrups of senega, or ipecacuanha, or antimony; the latter is best fitted for those cases in which there is much fever, and in many instances no inconvenience follows,—in others the stomach rejects all remedies of this class. There is, in fact, no expectorant used in the treatment of bronchitis which may not occasionally be given in phthisis; even the balsam of copaiva is sometimes of great benefit when there is much chronic bronchitis and but little fever or gastric irritability. In most cases, however, it is much too irritating.

Venesection is rarely requisite in pneumonia attending consumption, and is almost never indicated in bronchitis. Cupping is, however, of signal benefit in the treatment of both inflammations. Pneumonia in most cases does not need any other remedy; where an internal medicine is necessary, minute doses of tartarized antimony are much to be preferred to large ones, or to mercurials.

The treatment of pleurisy is more difficult.

The intimate relation of this disease with phthisis has already been explained, and it seems a matter of more urgent necessity to remove it completely at as early a period as possible. For this purpose the usual antiphlogistic measures are indicated at the beginning of the disease, with small doses of antimony and opium, or Dover's powder, or one of these remedies combined with digitalis, according to the strength of the patient. After the acute stages have passed off, the surest remedy is the repeated application of small blisters to the affected part from two to three inches square, so as to keep a continual counter-irritation, by changing the place of their application, and renewing them as often as they heal. Large blisters are better adapted to the acute than the chronic forms of pleurisy. It is often a question whether mercurials should be given under these circumstances; it is true that they produce an injurious effect upon phthisis proper; but when the inflammation of the serous membranes predominates very much over the scrofulous or tuberculous type peculiar to the disease, mercury may be given with discretion. That is, the circumstances proper for its employment are almost limited to the pleurisy of commencing phthisis; it does not seem to matter much whether the pleurisy be of that variety in which the tubercles are developed in the serous membrane, or whether they are formed afterwards in the substance of the lung. If we decide upon giving mercurials, they must be limited to very small doses, and should be given for a short time only, never producing ptyalism.

The other intercurrent inflammations must be treated as the same disorders would be if in an uncomplicated state. But gastritis and hæmoptysis, or tracheitis, require a passing notice. If the gastritis occur in a healthy individual in whom phthisis afterwards declares itself, there is nothing special in its management, but it often occurs in those who have previously offered the signs of a scrofulous diathesis, and is then difficult to treat. We must then look to the constitutional character of the disorder, and must place the patient upon general alteratives, especially the hygienic, as a sea-voyage or a journey, while we resort to the usual treatment for dyspepsia.

Laryngitis is often a mere symptom of phthisis; and if it occur in its advanced stage, opiates will scarcely succeed in palliating the distress which is caused by it; the difficulty of deglutition, and the uneasy feeling at the throat, often constitute one of the most disagreeable symptoms of advanced phthisis. But the commencing laryngitis is sometimes arrested readily enough when no tubercles are yet developed. Repeated, but small applications of leeches, followed by frictions with iodine ointment, and the internal use of the solution, are the most important means. A blister behind the neck is much less certain; sometimes it is applied in front of the larynx, but little benefit results from it except in the early stages. I have not used nor seen employed the cauterization of the larynx, by injecting a solution of nitrate of silver into it from a small syringe more than half full of air, so as to break the little stream into numerous fine drops. Dr. Trousseau gives some favourable accounts of its success; but I am not inclined to think that it is applicable to many cases. The pharyngitis which sometimes precedes phthisis, is more easy of cure, and requires local alteratives with a general tonic treatment. When the disease is nearly removed, cold alteratives of the neck and upper parts of the breast are the best means for preventing its return.

There are many symptoms in phthisis which are not connected with proper inflammations, but may reach a sufficient degree of intensity to require special treatment. These are the diarrhœa, hectic fever, and night sweats. Diarrhœa and dysentery often result from proper inflammation of the bowels, and are then nearly similar to the same disease as it occurs under ordinary circumstances. But diarrhœa assumes two other forms; it may be the proper tuberculous diarrhœa, which follows the softening of this substance in the follicles of the intestines, especially the glands of Peyer, or the colliquative diarrhœa, which occurs late in the disease, and sometimes carries the patient off very rapidly. The former variety of diarrhœa may be palliated by small doses of opiates; the latter requires the same treatment, but with less prospect of success; for when colliquative diarrhœa supervenes in the last stage of phthisis, it is almost always fatal. The astringents may be advantageously combined with opiates, especially kino, and given in small quantities, if the tongue is not dry and red, and they are not productive of gastric uneasiness.

If the diarrhœa is moderate, and the pectoral symptoms have abated upon its occurrence, it is better to abstain from any active medicines, as the discharge is then to a great extent a natural drain, and more mischief would follow from its repression than could be compensated for by the temporary relief of the patient. At the beginning of the diarrhœa, small doses, as two or three drachms of the spiced syrup of rhubarb, will often relieve it.

The sweats, which are so frequent in phthisis, are extremely exhausting to the patient, and sometimes require special remedies. The sweating is sometimes connected with the irritative form of early phthisis, or follows the hectic, which occurs only in the advanced cases. In other cases the sweating is a termination of a febrile paroxysm, and is in itself a mode of relief to the patient; but as it is a cause of great depression, and often prevents sleep, we are often compelled to resort to some efforts to check it. Several external applications have been proposed with this view, such as bathing the skin with a solution of alum, or some other astringent application. They sometimes succeed; but any external application intended to suppress what may be considered as a natural discharge, is fraught with danger; and whether this be sweating or the secretion from a cutaneous eruption, it should, if possible, be avoided. It is much better to use no external means, other than rendering the clothes as light during the period of fever as they can be made with safety to the patient. Another useful means of moderating the night sweat, is anticipating it. That is, the patient may take a hot pediluvium early in the evening, while he is still labouring under fever, and then go immediately to bed. The sweat will generally follow, and he should rise as soon as it diminishes and change his linen, and, if possible, the sheets of the bed. In this way he is almost sure of obtaining some hours of refreshing sleep. This remedy, however, like all others under similar circumstances, will fail after a time. The internal remedies used for the same purpose are notoriously uncertain. In fact, their administration is almost entirely empirical, and the most opposite medicine will sometimes succeed. That is, a remedy which produces a certain action upon the secretion of the gastric mucous membrane has generally a reciprocal action upon the cutaneous surface, and very different substances applied to the gastric membrane have the power of so modifying the condition of the whole body, that sweating is for a time suppressed. The most used of these remedies are the acids and alkalies, especially the former. The nitric or sulphuric acids are generally preferred to any other, especially the elixer of vitriol or aromatic sulphuric acid; this should be given in doses of from ten to twenty drops two or three times daily, either in some sweetened water, or in an infusion of the bark of wild cherry. Sometimes this remedy is disagreeable to the stomach, and produces various ill-defined sensations; it should then be discontinued, and a remedy of an opposite kind resorted to. The alkalies often answer well in such cases, especially lime water, with milk, in various proportions, as may be found to suit best with the stomach of the patient. This combination may be taken with some simple biscuit as a suitable article of food, when the stomach digests with difficulty. It should not, however, be used merely as an article of food, but be taken at different times throughout the day. Although it may seem singular that alkalies and acids are both of occasional benefit in the treatment of phthisis, yet it is what is often observed in ordinary cases of dyspepsia, in which the condition of the membrane of the stomach is not very dissimilar to that in phthisis, and the same causes which render these different remedies of service in the former case, appear to do so in the latter. At least this is the most reasonable explanation.

The chills of hectic fever are often extremely severe, and occur with such regularity that the sulphate of quinine has naturally enough been proposed as a remedy. Given in doses of from two to six grains before the expected chill, it will sometimes arrest it; in other cases it fails entirely, and is rather irritating to the patient than the contrary. In most cases the best treatment is to palliate the hectic by simple attention to warmth and other hygienic circumstances at the time of the paroxysm.

